# Brief reactive psychosis….again! - Clinical case report

**DOI:** 10.1192/j.eurpsy.2023.2217

**Published:** 2023-07-19

**Authors:** A. R. Costa, S. Jesus, M. Almeida, C. Vicente

**Affiliations:** Department of psychiatry and mental health, Baixo Vouga Hospital Centre, Aveiro, Portugal

## Abstract

**Introduction:**

Brief psychotic disorder according to the DSM-5 is a condition of sudden onset lasting less than 1 month followed by complete remission with possible future relapses, characterized by the development of psychotic conditions. The duration of the illness is a differentiating factor from other disorders such as schizophreniform psychosis or schizophrenia. When there is a stressful event at the origin of the psychotic symptomatology, it is also called brief reactive psychosis. The pathophysiology of BPD is not known, especially given the extremely low incidence of the disorder. This condition most often affects people in their 20s, 30s, and 40s, and its higher prevalence among patients with personality or mood disorders may suggest an underlying biological or psychological susceptibility that may have some genetic influence.

**Objectives:**

To describe the main diagnostic considerations, clinical manifestations, treatment, prognosis and prevention of brief reactive psychosis through the description of a clinical case that developed two episodes of brief reactive psychosis in a period of 1 year and to emphasize the importance of maintaining treatment for a period of suitable time.

**Methods:**

Case report and literature search with the terms: brief reactive psychosis, psychosis, neuroleptic, stressor event.

**Results:**

We describe the clinical case of a 29-year-old woman, born in S. Tomé and Príncipe, previously healthy, with no personal or family history of mental illness, who had her first brief reactive episode after coming to Portugal. With the introduction of the 2nd generation antipsychotic, paliperidone, there was a substantial improvement in the condition, however, with the development of side effects having subsequently abandoned the treatment. About 1 year after starting work in Portugal, she develops a new event, a new psychotic episode, with characteristics of a brief psychotic disorder.

**Conclusions:**

It is extremely important to alert patients to the possible side effects of drugs, as well as those who experience a brief psychotic episode, which are the risk factors and the need to comply with treatment in order to avoid a new relapse.

**Disclosure of Interest:**

None Declared

